# Design of 3D-printed prostheses for reconstruction of periacetabular bone tumors using topology optimization

**DOI:** 10.3389/fbioe.2023.1289363

**Published:** 2023-12-05

**Authors:** Jiazhuang Zhu, Jianping Hu, Kunpeng Zhu, Xiaolong Ma, Yongjie Wang, Enjie Xu, Zhen Huang, Yurun Zhu, Chunlin Zhang

**Affiliations:** Department of Orthopedic Surgery, Institute of Bone Tumor, Shanghai 10th People’s Hospital Affiliated to Tongji University, Tongji University School of Medicine, Shanghai, China

**Keywords:** 3D-printed prostheses, periacetabular bone tumors, topology optimization, finite element analysis, porous structure, clinical outcomes

## Abstract

**Background:** Prostheses for the reconstruction of periacetabular bone tumors are prone to instigate stress shielding. The purpose of this study is to design 3D-printed prostheses with topology optimization (TO) for the reconstruction of periacetabular bone tumors and to add porous structures to reduce stress shielding and facilitate integration between prostheses and host bone.

**Methods:** Utilizing patient CT data, we constructed a finite element analysis (FEA) model. Subsequent phases encompassed carrying out TO on the designated area, utilizing the solid isotropic material penalization model (SIMP), and this optimized removal area was replaced with a porous structure. Further analyses included preoperative FEA simulations to comparatively evaluate parameters, including maximum stress, stress distribution, strain energy density (SED), and the relative micromotion of prostheses before and after TO. Furthermore, FEA based on patients’ postoperative CT data was conducted again to assess the potential risk of stress shielding subsequent to implantation. Ultimately, preliminary follow-up findings from two patients were documented.

**Results:** In both prostheses, the SED before and after TO increased by 143.61% (from 0.10322 to 0.25145 mJ/mm^3^) and 35.050% (from 0.30964 to 0.41817 mJ/mm^3^) respectively, showing significant differences (*p* < 0.001). The peak stress in the Type II prosthesis decreased by 10.494% (from 77.227 to 69.123 MPa), while there was no significant change in peak stress for the Type I prosthesis. There were no significant changes in stress distribution or the proportion of regions with micromotion less than 28 μm before and after TO for either prosthesis. Postoperative FEA verified results showed that the stress in the pelvis and prostheses remained at relatively low levels. The results of follow-up showed that the patients had successful osseointegration and their MSTS scores at the 12th month after surgery were both 100%.

**Conclusion:** These two types of 3D-printed porous prostheses using TO for periacetabular bone tumor reconstruction offer advantages over traditional prostheses by reducing stress shielding and promoting osseointegration, while maintaining the original stiffness of the prosthesis. Furthermore, *in vivo* experiments show that these prostheses meet the requirements for daily activities of patients. This study provides a valuable reference for the design of future periacetabular bone tumor reconstruction prostheses.

## 1 Introduction

Limb-salvage surgery, including tumor excision and biological reconstruction, has found extensive application in pelvis tumor therapy. This field encompasses procedures such as massive pelvis allografts (Matejovsky et al., 2006; Ayvaz et al., 2014) and extracorporeally irradiated autografts (Wafa et al., 2014). Contemporary prosthetic options for reconstruction comprise ice-cream cone prostheses (Barrientos-Ruiz et al., 2017; Fujiwara et al., 2021), saddle-shaped prostheses (Aboulafia et al., 1995; Kitagawa et al., 2006; Danışman et al., 2016), modular hemipelvis prostheses (Ji et al., 2010; Li et al., 2022), and three-dimensionally printed (3D-printed) prostheses (Wang et al., 2020; Zhu et al., 2021; Guo et al., 2022). Most of these biological reconstruction techniques have yielded encouraging outcomes in subsequent assessments. Nevertheless, when addressing bone tumors situated in intricate locales, 3D-printed prostheses offer enhanced precision and temporal efficiency. Consequently, its application within the realm of pelvis tumor treatment has observed a progressive upsurge (Wang et al., 2020; Guo et al., 2022; Zhu et al., 2022).

Ti-6Al-4V stands as a prevalent material for pelvis prostheses that is characterized by a Young’s modulus of 110 GPa. In contrast, cortical bone exhibits a Young’s modulus typically below 30 GPa, while the modulus of cancellous bone falls below 2 GPa (Immel et al., 2021; Kang et al., 2021; Wu et al., 2021). The marked dissimilarity in mechanical attributes between the pelvis and the prostheses can instigate stress shielding, leading to complications such as periprosthetic bone resorption, aseptic loosening of prostheses, and periprosthetic fractures (Kitamura et al., 2005; Arabnejad et al., 2017; Wu et al., 2021; Zhou et al., 2022). Earlier investigations of this topic have demonstrated that adaptations in geometric shapes, materials, or the integration of porous frameworks can mitigate prosthetic rigidity, thus tempering stress shielding (Glassman et al., 2006; Iqbal et al., 2019; Vance et al., 2019; Zhou et al., 2022; Rana et al., 2023). Among these strategies, the incorporation of porous architectures not only bestows a diminished elastic modulus but also fosters biological activity, enhancing the amalgamation of prostheses with host bone and promoting soft tissue adherence (Chen et al., 2018; Lv et al., 2021). Consequently, the integration of porous structures has emerged as a widely embraced technique in recent years to address stress incongruence between prostheses and host bones.

A recent review highlighted the utilization of uniform, graded, and optimized strategies in the design of porous prostheses. Among these strategies, optimizing porous designs for prosthetic mechanical performance using 3D reconstructions with CT scans has shown elevated reliability (Safavi et al., 2023). Notably, within the commonly used optimization approaches, TO holds prominence. Initially, rooted in industrial domains such as the aerospace and automotive sectors, TO aims to attain optimal material distribution under defined constraints (Yang and Chahande, 1995; Zhu et al., 2016). In the medical realm, 3D reconstruction and TO have found diverse applications spanning mandible bone (Peng et al., 2021), spine (Kang et al., 2021), pelvic (Iqbal et al., 2019; Giudice et al., 2020), and hip prostheses (Kladovasilakis et al., 2020). Nevertheless, it remains unexplored in the context of porous design for 3D-printed prostheses catering to periacetabular bone tumor reconstruction and subsequent clinical application.

Therefore, this study introduces an innovative, complete, and comprehensive TO method to determine the area to add porous structure to pelvic prostheses. This method is dual validated via computer simulations and postoperative assessments. The objective is to design prostheses possessing porous structures for periacetabular bone tumor reconstruction that mitigate the likelihood of stress shielding and concurrently foster osseointegration between the prostheses and the host bone.

## 2 Materials and methods

**FIGURE 1:**
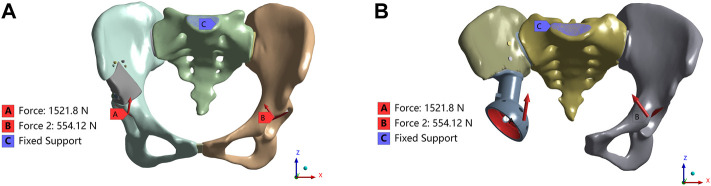
Load and boundary conditions in the FEA model. The coordinate system is established in Mimics software based on the CT data. The *X*-axis is oriented vertically along the sagittal plane of the CT, extending from right to left. Similarly, the *Y*-axis passes vertically through the coronal plane of the CT, from front to back. Lastly, the *Z*-axis traverses the cross-sectional plane of the CT vertically, from bottom to top **(A)** Type I; **(B)** Type II.

In June 2020 and June 2021, our hospital treated two patients with periacetabular bone tumors, including one case of benign bone tumor and one case of Enneking type II + III bone tumor. Both patients had an expected lifespan of over 6 months and willingly accepted the potential risks associated with using 3D-printed personalized prostheses for postresection reconstruction. To deliver this, we designed two personalized prostheses for bone defect reconstruction after resection of different periacetabular bone tumors. Type I for limited bone defect caused by resection due to benign bone tumors, and Type II for hemipelvectomies of Enneking II + III bone tumors (Figure 1).

### 2.1 Establishment of the FEA model

The patient’s CT data were imported into Mimics software (version 21.0, Materialise, Leuven, Belgium) in DICOM format to generate a 3D model of the pelvis. Subsequently, the pelvis model was exported as a stereolithography (STL) file. This STL file was then imported into Geomagic Wrap software (version 2017, USA) for patching and grid construction and subsequently exported as a Step—AP203 (STEP) file. The obtained STEP file was further imported into SOLIDWORKS software (version 2020, Dassault Systemes, France), where the sacroiliac joints, symphysis pubis, screws, prosthesis, and contact plate (if necessary) were generated. To facilitate FEA, the screws were simplified as cylindrical shapes. After assembly, all parts of the model were again exported in STEP format.

These STEP files were subsequently imported into HyperMesh software (version 2020, Altair Engineering Inc., USA) to generate 4-node linear tetrahedron elements (C3D4). In addition, we conducted a mesh sensitivity analysis and found that the stresses gradually increased when the number of meshes was gradually increased, and the effect of the mesh on the results was considered to be acceptable (<5%) when the mesh size was less than 1 mm; thus, the mesh size was set to 1 mm. The model was then exported in Ansys Preprocessor (CDB) format. Within the Mimics software, material properties for the pelvis were assigned based on Hounsfield units, bone density, and elastic modulus using Formulas (1) and Formulas (2) (Iqbal et al., 2019; Moussa et al., 2020). These resulting files were also exported in CDB format.

Finally, these generated CDB files were integrated into ANSYS software (versions 2020R1 and 2022R1, Canonsburg, Pennsylvania, USA) utilizing the external model module, and subsequently, a static structural module was created to establish necessary connections. Homogeneous material properties were assigned to other components, as shown in Table 1 (Shi et al., 2014; Iqbal et al., 2019; Moussa et al., 2020).

$$\rho =6.9141e^{-4}\times HU+1.026716$$ (1)

$$E=2017.3\times \rho^{2.46}$$ (2)

**TABLE 1:** Material properties of entities.

Entity	Material	Elastic modulus (MPa)	Poisson’s ratio
Pelvis	Inhomogeneous		0.3
Sacroiliac joint	Homogeneous	54	0.4
Symphysis pubis	Homogeneous	5	0.45
Prostheses	Ti-6Al-4V	110000	0.3
Contact plate (solid)	Ti-6Al-4V	110000	0.3
Screw	Ti-6Al-4V	110000	0.3

The load and boundary conditions are depicted in Figure 1. The full constraint was applied to the superior surface of the sacrum, while the magnitudes and directions are shown in Table 2, with reference to the peak contact forces applied through the bilateral acetabulum during normal walking in humans (Iqbal et al., 2019). We assumed a frictionless face-to-face contact at the prosthesis/connecting plate-host bone interface to simulate the initial non-fusion state of prostheses within the bone. Subsequently, frictional contact with a coefficient of 0.2 was employed for the screws and pelvis (Bulaqi et al., 2015; Wang et al., 2020). Other parameters were maintained at their default values within the software. Key parameters for assessing stress shielding and prosthesis-host bone osteointegration include stress distribution, maximum stress, relative micromotion, and SED. Relative micromotion outcomes were determined through the application of the Contact Tool. The SED values were computed using the User Defined Result feature, specifically the ENERGYPOTENTIAL/VOLUME function.

**TABLE 2:** The force of the pelvis during normal walking.

Application region	F_x_(N)	F_y_(N)	F_z_(N)	Combined forces(N)^a^
Right acetabulum	230.18	-164.39	1495.24	1521.8
Left acetabulum	-325.45	-39.26	446.75	554.12

^a^ as shown in Figure 1.

### 2.2 TO, the addition of porous structures, fabrication, and surgery

The structural optimization module was added to ANSYS (version 2020R1, Canonsburg, Pennsylvania, USA), and the FEA outcomes were integrated into the module. For optimization, Type I prostheses and Type II connecting plates were designated as the target area, utilizing the SIMP method with a penalty coefficient of 3. The optimization objective sought to minimize compliance while upholding volume fraction constraints of 20% (Type I) or 10% (Type II). Default parameters were maintained for other configurations. The resulting optimization outputs were exported in STEP file format and subsequently imported into SOLIDWORKS.

Within SOLIDWORKS, Boolean operations were employed to fix undesirable regions through geometric reconstruction adjustments to guarantee both manufacturability and safety. Subsequently, using Magics (version 21.0, Materialise, Leuven, Belgium), the areas that were optimized and removed underwent substitution with a porous architecture featuring a pore size of 600 μm and a porosity of 70%. We assumed that prostheses with the addition of porous structures would have better stress transfer, maintaining a similar stress distribution while promoting bone ingrowth. Furthermore, pertinent elastic modulus information for a porous structure characterized by the same pore size (600 μm) and porosity (70%) was obtained from relevant literature sources (Bartolomeu et al., 2019b; Wang et al., 2020a). A subsequent comparison was conducted between these data and the corresponding outcomes derived from the FEA.

Upon completion of the design phase, manufacturing was carried out by Shengshi Co., Ltd. The prosthesis fabrication was carried out by the SLM technique using Ti-6Al-4 V powders with 20–53 μm particle size and 50 μm layer thickness with a scanning speed of 1300 mm/s. Excess powder particles were cleaned by compressed air and ultrasound. The prosthesis was kept at 800°C for 2 h with natural cooling under argon protection. The surgical procedures were performed by the same surgeon (CL Zhang), and these procedures include a single case of Type I and a single case of Type II.

### 2.3 Postoperative FEA and follow-up

To counteract potential modelling-induced biases in pre- and postoperative computer simulations, a simplified modelling approach is employed based on the patient’s postoperative CT data, which facilitates quick determination of the relative placement of postoperative pelvic prostheses. Subsequently, adjustments are made to the positions of the pelvis, prostheses, and screws in the preoperative model to achieve optimal alignment with the postoperative model while keeping all other parameters constant. This is followed by validation of the postoperative computer simulation.

Moreover, a comprehensive postoperative follow-up is undertaken to validate the derived results. This follow-up involves regular physical examinations and imaging assessments after discharge, which occur every 3 months during the first postoperative year, every 6 months between the first and second years, and annually thereafter. The primary aim of these assessments is to evaluate osseointegration. Additionally, the patients’ resting pain and limb functionality are evaluated using MSTS scores.

### 2.4 Data analysis

Analysis was conducted using SPSS (Version 25, IBM Corporation, Armonk, NY, USA). The SED of prostheses before and after TO underwent non-parametric testing. The chi-square test assessed relative micromotion at the bone-prosthesis interface. Statistical significance was established at *p* < 0.05.

## 3 Results

The number of elements and nodes of the FEA models used for both types of prostheses are shown in Table 3.

**TABLE 3:** The number of nodes and elements of the FEA models used for the two types of prostheses.

Model	Node	Element
Type I before TO	422552	2035329
Type I after TO	430027	2065861
Type I after surgery	425125	2043980
Type II before TO	428340	2065082
Type II after TO	428537	2065577
Type II after surgery	429147	2068852

### 3.1 Type I

**FIGURE 2:**
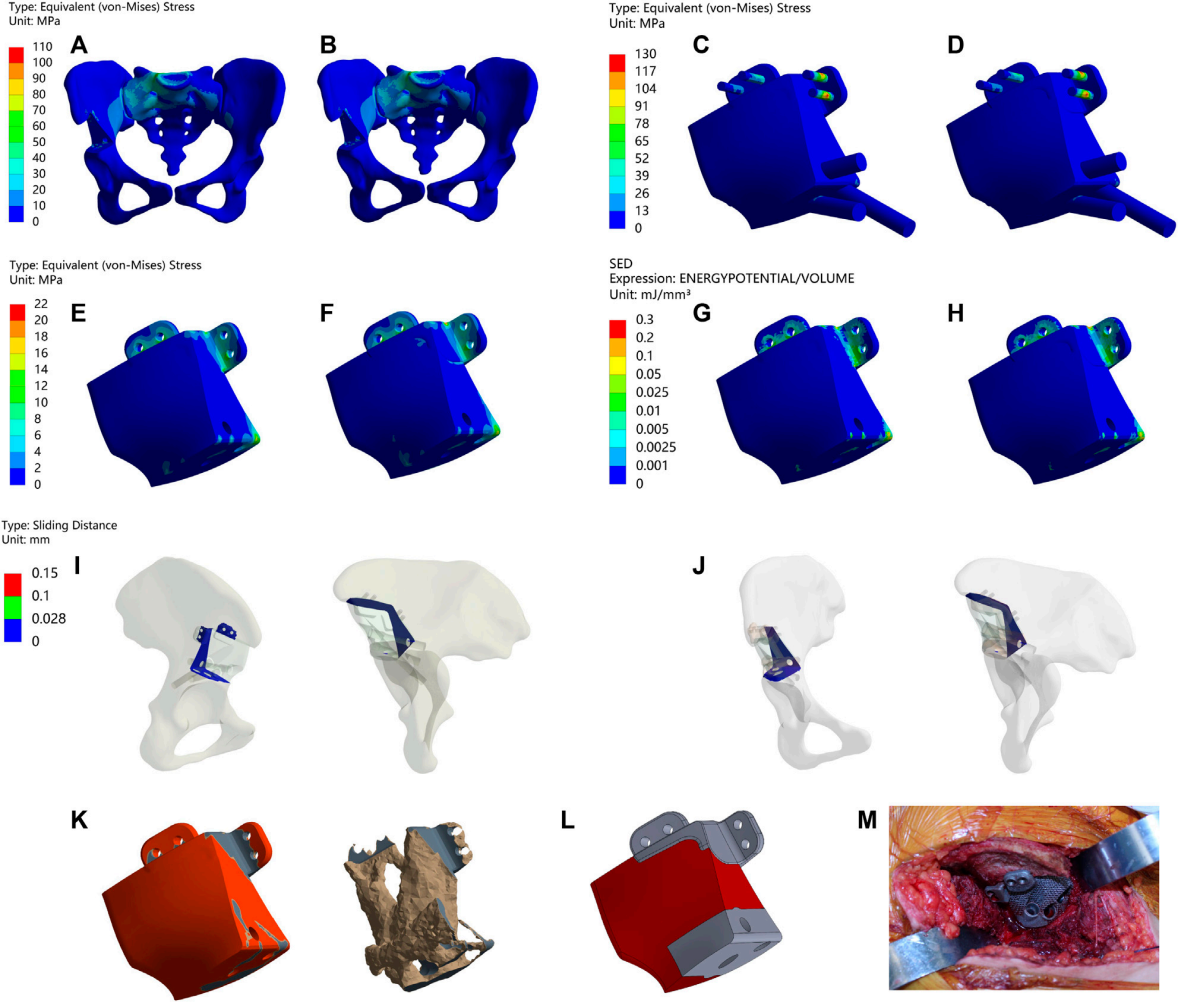
Results of Type I before and after TO **(A,B)** Stress on the pelvis before and after TO **(C,D)** Stress on the prosthesis and screws before and after TO **(E,F)** Stress on the prosthesis before and after TO. **(G,H)** SED of the prosthesis before and after TO **(I,J)** Relative micromotion of bone-prosthesis interface before and after TO. **(K)** TO results, where the bright color is the part removed after optimization and the other colors are the part of retained **(L)** The final design, where the bright color is porous structure and the other color is solid structure. **(M)** Installation of prosthesis.

As shown in Figure 2 and Table 4, Type I reconstruction prosthesis demonstrates consistent peak stress levels in the pelvis, regardless of TO. Both before and after TO measurements yield a peak stress of 107.11 MPa, with a notable concentration in the affected side ilium and adjacent sacrum. Moreover, insignificant differences in peak stress between the prosthesis and screws are observed before and after TO, measuring 125.46 and 123.46 MPa, respectively. Nevertheless, the peak stress of the prosthesis experiences a 27.296% increase (from 16.896 to 21.508 MPa) after TO, with a pronounced concentration around the four fixed screws positioned above the prosthesis. Additionally, the peak SED of the prosthesis rises significantly by 143.61% after TO, escalating from 0.10322 to 0.25145 mJ/mm³ (*p* < 0.001). Subsequently, the analysis reveals an augmentation in maximum relative micromotion at the bone-prosthesis interface, elevating the measurement from 27.516 to 30.386 μm. Despite this trend, no statistical significance is observed, and the proportion of regions experiencing micromotion less than 28 μm marginally decreases from 100% to 99.846% (*p* = 0.133).

**TABLE 4:** Maximum values and percentage of relative micromotion below 28 μm for Figure 2.

Figure 2	Data types	Result
A&B	MAX	107.11 and 107.11
C&D		125.46 and 123.46
E&F		16.896 and 21.508
G&H		0.10322 and 0.25145^a^
I&J		0.027516 and 0.030386
I&J	less than 28 µm	100% and 99.846% ^ns^

^a^ *p* < 0.001; ^ns^, not significant.

**FIGURE 3:**
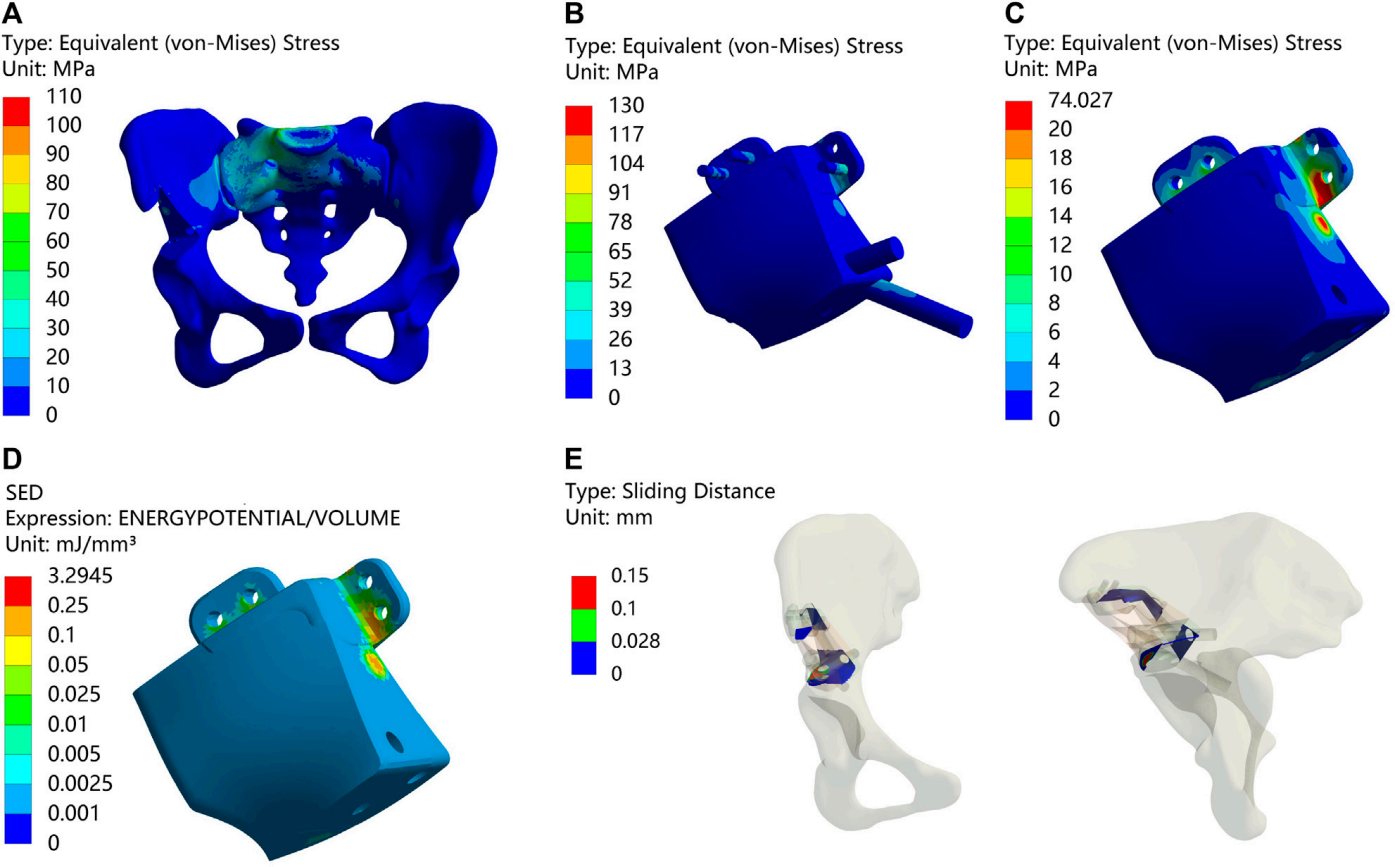
Results of Type I after surgery **(A)** Stress on the pelvis. **(B)** Stress on the prosthesis and screws **(C)** Stress on the prosthesis. **(D)** SED of the prosthesis. **(E)** Relative micromotion of bone-prosthesis interface.

Postsurgical alterations consisted of removing two screws originally planned for the procedure and adjusting the position of the prosthesis. As a result, distinct outcomes are displayed in Figure 3 and Table 5. The peak stress in the pelvis following surgery slightly decreased compared to the preoperative plan, measuring 96.216 MPa, while maintaining stress concentration in the affected side ilium and adjacent sacrum. Strikingly, both the peak stress and SED of the prostheses after surgery substantially surpassed the preoperative plan, measuring 74.027 MPa and 3.2945 mJ/mm³, respectively. Remarkably, stress concentration zones coincide with the placement of the three fixed screws above the prosthesis. Despite the noteworthy maximum relative micromotion at the bone-prosthesis interface postsurgery, reaching 124.46 μm, a relatively high proportion of regions (90.175%) still exhibit micromotion less than 28 μm.

**TABLE 5:** Maximum values and percentage of relative micromotion below 28 μm for Figure 3.

Figure 3	Data types	Result
A	MAX	96.216
B		74.027
C		74.027
D		3.2945
E		0.12446
E	less than 28 µm	90.175%

### 3.2 Type II

**FIGURE 4:**
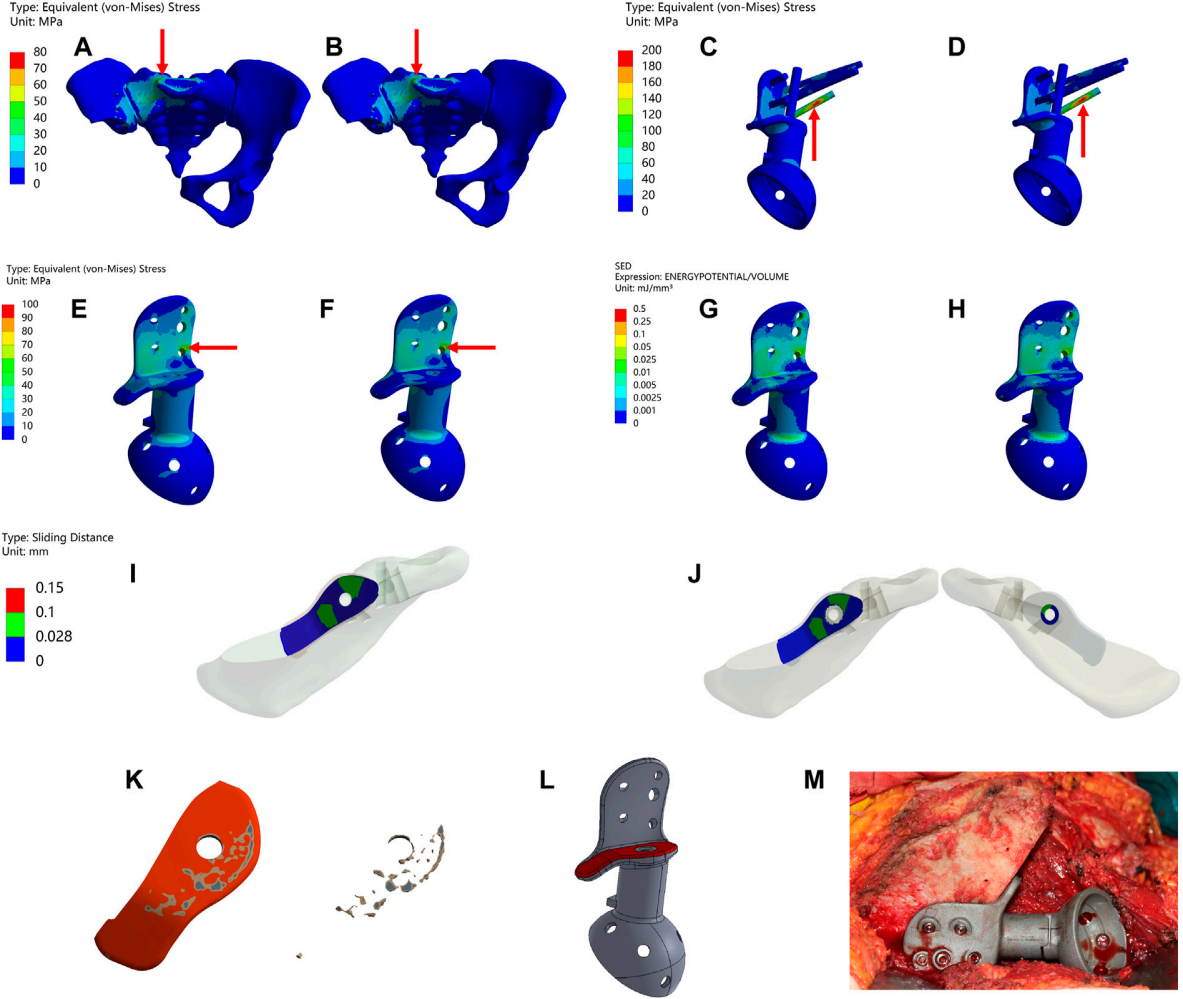
Results of Type II before and after TO (The arrows point to the maximum value) **(A,B)** Stress on the pelvis before and after TO. **(C,D)** Stress on the prosthesis and screws before and after TO **(E,F)** Stress on the prosthesis before and after TO. **(G,H)** SED of the prosthesis before and after TO **(I,J)** Relative micromotion of bone-prosthesis interface before and after TO. **(K)** TO results, where the bright color is the part removed after optimization and the other colors are the part of retained **(L)** The final design, where the bright color is porous structure and the other color is solid structure. **(M)** Installation of prosthesis.

Figure 4 and Table 6 illustrate the results of the type II reconstruction prosthesis. The peak stress in the pelvis decreased by 10.494% (from 77.227 to 69.123 MPa) before and after TO, with stress concentration observed in the affected side ilium and adjacent sacrum. Negligible differences were observed in the peak stresses of both prosthesis and screws before and after TO, measuring 197.03 and 197.10 MPa, respectively. Likewise, minimal changes occurred in the peak stress of the prosthesis before and after TO, measuring 98.567 and 98.555 MPa, respectively, with a relatively uniform stress distribution. Furthermore, the peak SED of the prosthesis significantly increased by 35.050% (from 0.30964 to 0.41817 mJ/mm³, *p* < 0.001). Despite a decrease in maximum relative micromotion between bone and prosthesis from 70.155 to 67.597 μm after TO, the proportion of areas with micromotion less than 28 μm slightly decreased from 79.232% to 79.077%, albeit this change was statistically insignificant (*p* = 0.922).

**TABLE 6:** Maximum values and percentage of relative micromotion below 28 μm for Figure 4.

Figure 4	Data types	Result
A&B	MAX	77.227 and 69.123
C&D		197.03 and 197.10
E&F		98.567 and 98.555
G&H		0.30964 and 0.41817^a^
I&J		0.070155 and 0.067597
I&J	less than 28 µm	79.232% and 79.077% ^ns^

^a^ *p* < 0.001; ^ns^, not significant.

**FIGURE 5:**
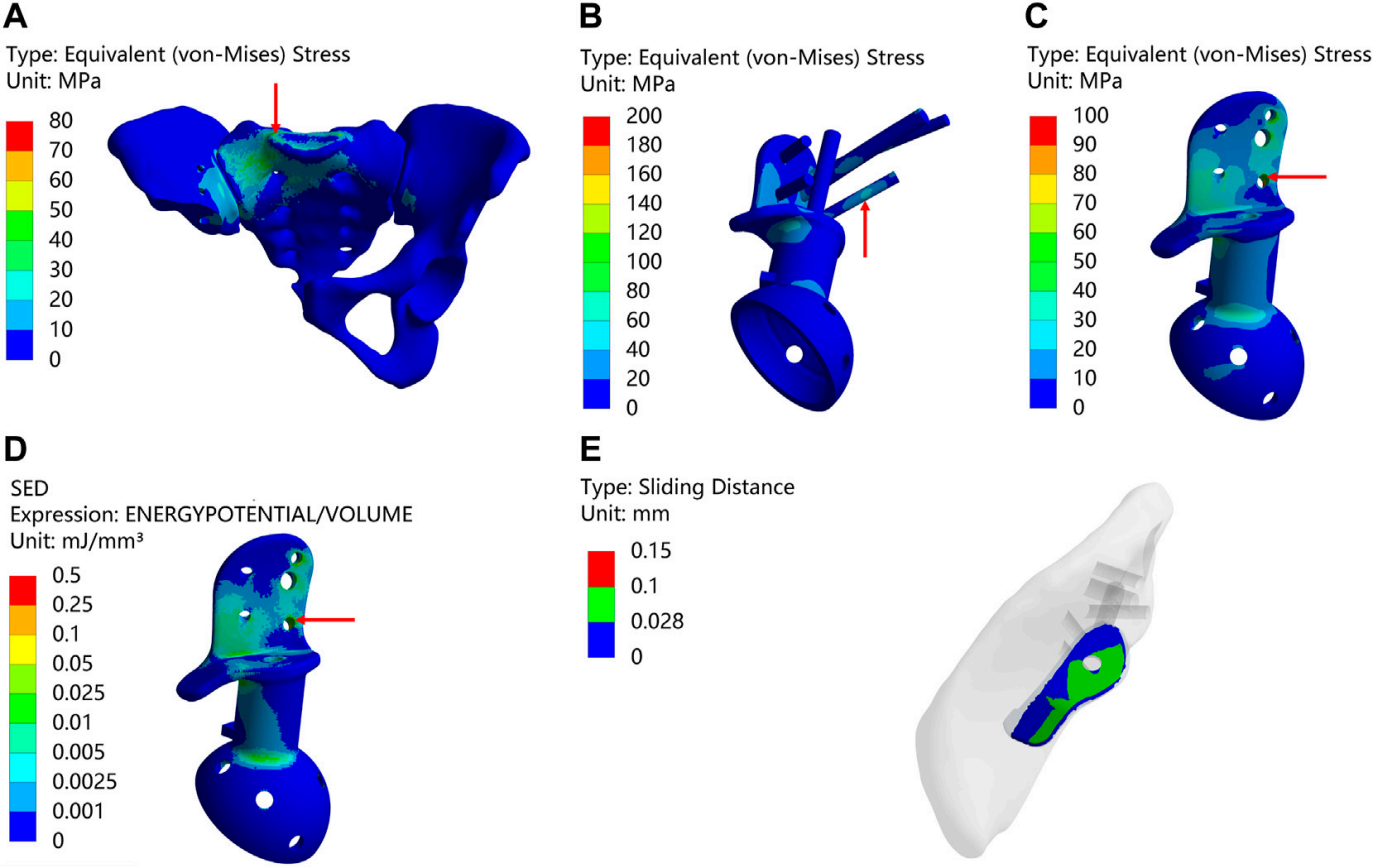
Result of Type II after surgery (The arrows point to the maximum value) **(A)** Stress on the pelvis. **(B)** Stress on the prosthesis and screws **(C)** Stress on the prosthesis. **(D)** SED of the prosthesis. **(E)** Relative micromotion of bone-prosthesis interface.

Following the surgical procedure, adjustments were made to the relative positions of the prosthesis and screws compared to the preoperative planning. As a result, distinct outcomes are displayed in Figure 5 and Table 7. The peak stress on the pelvis after surgery, 72.914 MPa, was similar to the preoperative plan, with stress concentration observed in the affected side ilium and adjacent sacrum. Subsequently, the peak stress and SED of the prosthesis after surgery were recorded as 109.15 MPa and 0.11781 mJ/mm³, respectively, which were lower than the preoperative planning. Moreover, the maximum relative micromotion between bone and prosthesis after surgery measured 100.94 μm, with only 50.018% of areas experiencing micromotion less than 28 μm.

**TABLE 7:** Maximum values and percentage of relative micromotion below 28 μm for Figure 5.

Figure 5	Data types	Result
A	MAX	72.914
B		109.15
C		109.15
D		0.11781
E		0.10094
E	less than 28 µm	57.018%

### 3.3 Postoperative follow-up

**FIGURE 6:**
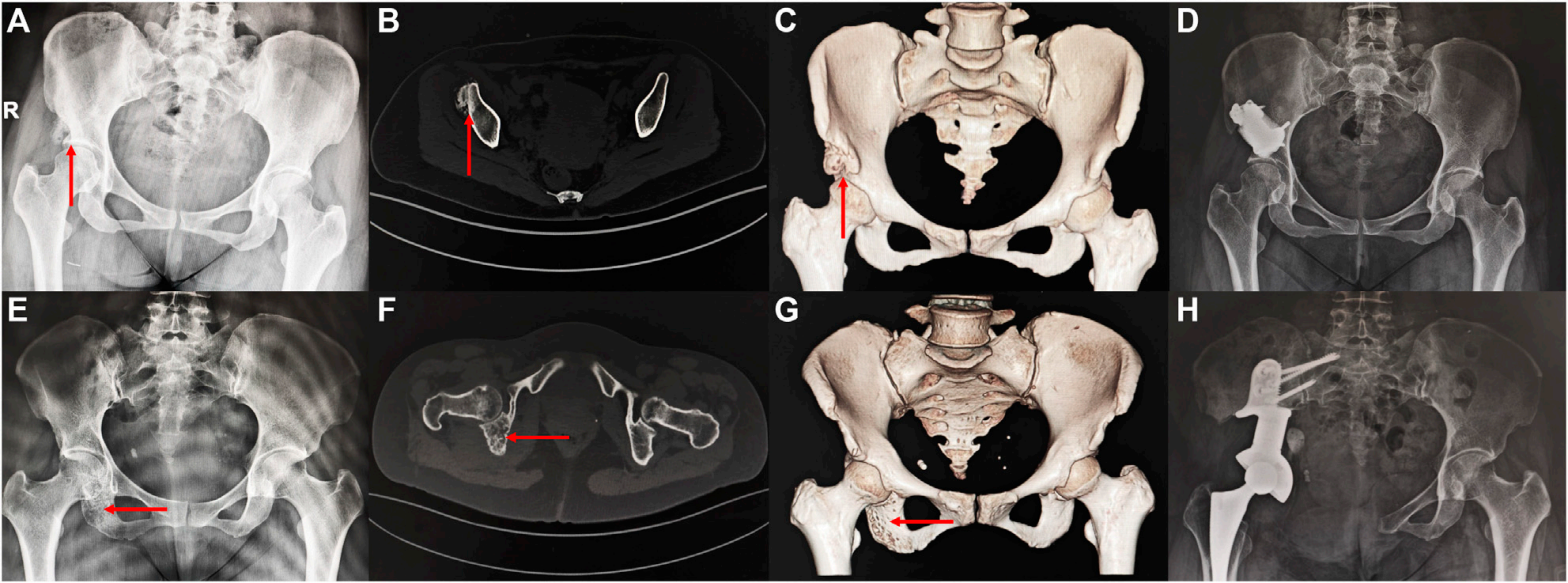
Imaging of the patient preoperatively and postoperatively **(A–D)** a 46 years old female patient diagnosed with osteoidostema; **(E–H)** a 49 years old female patient diagnosed with leiomyosarcoma.

The information about the two patients is shown in Figure 6. Both patients achieved primary wound healing within 2 weeks without any major complications. However, the patient who underwent Type-I prosthesis reconstruction experienced numbness of the lateral cutaneous nerve of the thigh as a postoperative complication related to the surgical approach. The length of hospital stay for Type-I prosthesis reconstruction was 7 days, while it was 16 days for Type-II prosthesis reconstruction. The time needed for out-of-bed ambulation after surgery was 24 days for Type-I prosthesis reconstruction and 36 days for Type-II prosthesis reconstruction. The follow-up period was 36 months for Type-II prosthesis reconstruction and 15 months for Type-I prosthesis reconstruction. By this time, both patients had returned to their daily work, and their MSTS scores at the 12th month after surgery were both 100% (Supplement Materials).

## 4 Discussion

In this study, we conducted to design Type I and Type II pelvis prostheses with porous structures, which were subsequently implemented in patients. Through a combination of FEA and clinical outcomes, our research has demonstrated the efficacy of these optimized prostheses to effectively reduce the risk of stress shielding and promote prosthesis-host bone osseointegration.

There are six most common activities of the human body, including normal walking, single-leg standing, dual-leg standing, sitting down, ascending stairs, and descending stairs. Iqbal et al. (2019), designed four specific pelvis prostheses through multiobjective TO based on these six loading conditions. In the analysis and TO of hemipelvis prostheses, unilateral acetabulum or femoral head loading simulations for single-leg standing have been extensively used. For example, Zhou et al. (2022), applied loads of nearly 2000 N at the centre of the prosthetic acetabulum, and Moussa et al. (2020), applied similar loads to the femoral head, successfully conducting TO to obtain the stress distribution of the hemipelvis prostheses under extreme loading conditions. However, since this loading condition fails to encompass the entire pelvis structure, the resulting stress distribution may deviate from the actual physiological scenario. Furthermore, some studies have attempted to simulate pelvic loading during human standing by applying loads to the S1 surface of the sacrum, but most of these works only conducted FEA without incorporating TO. In contrast, our study performed a comprehensive assessment of stress distribution throughout the complete pelvis structure following prosthetic reconstruction. This was achieved by subjecting both acetabula to loads during normal walking, which represents the most common activity in the human activity cycle (Bergmann et al., 2001; Iqbal et al., 2019).

In both types of prostheses, the stress on the pelvis before and after TO is similar and lower than the yield strength of cortical bone (150 Mpa). The peak stress of screws is generally higher than that of prostheses but significantly lower than the yield strength (789–1013 Mpa) and fatigue limit (310–610 Mpa) of Ti-6Al-4V (Long and Rack, 1998; Iqbal et al., 2017; Dong et al., 2018). The similar stress in the prostheses before and after to suggests that the application of a porous structure has minimal impact on the peak stress of the overall prostheses. However, in the case of Type II, the optimized contact plate exhibits lower stress levels compared to the previous contact plate, indicating that a porous structure can effectively reduce the contact stress at the bone-prosthesis interface, which aligns with previous studies (Moussa et al., 2020).

SED is a significant indicator that is used to assess the risk of stress shielding in prostheses, with higher values indicating a reduced risk of stress shielding (Zhang et al., 2016; Wang et al., 2020; Zhou et al., 2022). In this study, the SEDs of Type I and Type II prostheses exhibited increases of 143.61% and 35.050%, respectively. These heightened SED values demonstrate that the incorporation of TO and a porous structure result in enhanced mechanical stimulation of the pelvis prostheses, leading to improved stress transmission during movement and reducing the risk of stress shielding and bone resorption, which can reduce impediments to bone growth and ultimately reduce the risk of prosthesis loosening (Lin et al., 2004; Liu et al., 2021; Zhang et al., 2021; Zhang et al., 2023).

The level of relative micromotion at the bone-prosthesis interface is a crucial indicator for evaluating bone growth. It is widely accepted that micromotion below 28 µm promotes bone growth, while micromotion exceeding 150 µm inhibits bone growth (Kienapfel et al., 1999; Zhao et al., 2018; Moussa et al., 2020; Zhou et al., 2022). Here, we assumed that there was no frictional contact at the bone-prosthesis interface, mimicking the situation where the prostheses were not fully integrated during the early postoperative period to achieve a more realistic level of relative micromotion. However, the results showed that although the proportion of relative micromotion below 28 μm was maintained at a high level, there was no difference before and after TO. These findings differ from prior research suggesting that topological optimization and porous structures can reduce the level of relative micromotion at the bone-prosthesis interface (Xue et al., 2022; Zhou et al., 2022).

Previous studies have shown that an appropriate porous structure (with a porosity of 70% and pore sizes ranging from 100–800 μm) at the bone-prosthesis interface promotes closer tissue contact, facilitates inwards growth, and maintains proper mechanical strength (Tsuruga et al., 1997; Chen et al., 2017; Bartolomeu et al., 2019a; Wang et al., 2020; Kang et al., 2021; Xue et al., 2022; Zhou et al., 2022). Based on these findings and in combination with published research (Wang et al., 2020; Wang et al., 2020; Xue et al., 2022; Zhou et al., 2022), we chose a porous structure with a porosity of 70% and a pore size of 600 μm. TO is a useful tool in orthopaedic prosthesis design (Wang et al., 2016; Wu et al., 2021), with applications in the pelvis (Iqbal et al., 2019; Moussa et al., 2020), limb bones (Rahimizadeh et al., 2018; Xue et al., 2022), and spine (Moussa et al., 2018; Moussa et al., 2020). While both methods have been widely applied in orthopedic prostheses, there have been few studies that combine them. For example, Zhou et al. (2022) performed TO and porous structure design on type I + II + III pelvis prostheses, resulting in higher SED and lower surface micromotion in the redesigned prostheses. Wang et al. (2020) achieved enhanced porous fusion cages through global-local topological optimization, leading to improved mechanical performance. To our knowledge, this is the first study that applies TO for porous design to obtain 3D-printed prostheses for reconstructing periacetabular bone tumours and applying them in the clinic. In this study, compared to fully solid prostheses, the prostheses after TO showed a significant increase in SED, while stress and relative micromotion remained at lower levels. The optimized contact plate reduced the contact stress at the bone-prosthesis interface, and postoperative follow-up evidence indicated that patients had successful osseointegration. This outcome suggests that the TO for the porous design method used in this study can reduce the risk of stress shielding and promote prosthesis-host bone osseointegration.

This study has some limitations to acknowledge. First, for computational purposes, we simplified the screw to a cylindrical shape and ignored the effects of muscles and ligaments, and this simplification in the model likely biased the FEA results. Although some studies (Dalstra and Huiskes, 1995; Iqbal et al., 2017) have suggested that the effect of muscles on stress is negligible, it is undeniable that muscle forces can lead to a more homogeneous distribution of stress in pelvic prosthesis models (Phillips et al., 2007), however, further research is needed to determine the extent to which this simplification affects the findings of this study. Second, our model only accounted for loads during normal walking and did not consider other common activities, such as walking up and down stairs or standing on one leg. As such, the results of the TO may not fully represent the stresses experienced by patients during all activities, which should be considered in future studies. Finally, in addition to preoperative simulation and postoperative numerical model validation, we did not conduct mechanical compression experiments to verify the mechanical performance of the prostheses. Additionally, although we applied it for clinical verification, and the results of the follow-up proved the reliability of the design method proposed in this paper, the results obtained from the clinical follow-up did not accurately reflect the results of this research method due to the small sample size, which also hindered the further generalization of this research method (Wang et al., 2020; Kang et al., 2021; Zhu et al., 2021). Therefore, future studies should address larger population groups with longer-term follow-ups.

## 5 Conclusion

This study utilized the TO method to determine the area of the porous structure of two types of 3D-printed pelvis prostheses for limited bone defects caused by limited resection due to benign tumors and hemipelvectomies of Enneking II + III bone tumors. Compared to solid prostheses, these two types of 3D-printed porous prostheses, which use TO for periacetabular bone tumor reconstruction, offer the advantages of reducing stress shielding and promoting osseointegration while maintaining the original stiffness of the prosthesis. Furthermore, *in vivo* experiments showed that these prostheses meet the requirements for suiting daily activities of patients. This study provides a valuable reference for the design and application of future periacetabular bone tumor reconstruction prostheses.

## Data Availability

The raw data supporting the conclusion of this article will be made available by the authors, without undue reservation.
